# Complexities of implementing Maternal and Perinatal Death Surveillance and Response in crisis-affected contexts: a comparative case study

**DOI:** 10.1186/s13031-024-00607-3

**Published:** 2024-07-16

**Authors:** Meighan Mary, Hannah Tappis, Elaine Scudder, Andreea A. Creanga

**Affiliations:** 1 International Health Department, Bloomberg School of Public Health, Johns Hopkins University, 615 N Wolfe St, Baltimore, MD 21205 USA; 2 Center for Humanitarian Health, Johns Hopkins University, Baltimore, MD USA; 3 International Center for Maternal and Newborn Health, Johns Hopkins Bloomberg School of Public Health, Baltimore, MD USA; 4 Jhpiego, Baltimore, MD USA; 5 International Rescue Committee, Washington, DC USA; 6 Department of Gynecology and Obstetrics, Johns Hopkins School of Medicine, Baltimore, MD USA

**Keywords:** Maternal mortality, Neonatal mortality, Surveillance, Death review, Maternal and perinatal death surveillance and response (MPDSR), Humanitarian

## Abstract

**Background:**

Maternal and Perinatal Death Surveillance and Response (MPDSR) systems provide an opportunity for health systems to understand the determinants of maternal and perinatal deaths in order to improve quality of care and prevent future deaths from occurring. While there has been broad uptake and learning from low- and middle-income countries, little is known on how to effectively implement MPDSR within humanitarian contexts – where disruptions in health service delivery are common, infrastructural damage and insecurity impact the accessibility of care, and severe financial and human resource shortages limit the quality and capacity to provide services to the most vulnerable. This study aimed to understand how contextual factors influence facility-based MPDSR interventions within five humanitarian contexts.

**Methods:**

Descriptive case studies were conducted on the implementation of MPDSR in Cox’s Bazar refugee camps in Bangladesh, refugee settlements in Uganda, South Sudan, Palestine, and Yemen. Desk reviews of case-specific MPDSR documentation and in-depth key informant interviews with 76 stakeholders supporting or directly implementing mortality surveillance interventions were conducted between December 2021 and July 2022. Interviews were recorded, transcribed, and analyzed using Dedoose software. Thematic content analysis was employed to understand the adoption, penetration, sustainability, and fidelity of MPDSR interventions and to facilitate cross-case synthesis of implementation complexities.

**Results:**

Implementation of MPDSR interventions in the five humanitarian settings varied in scope, scale, and approach. Adoption of the interventions and fidelity to established protocols were influenced by availability of financial and human resources, the implementation climate (leadership engagement, health administration and provider buy-in, and community involvement), and complex humanitarian-health system dynamics. Blame culture was pervasive in all contexts, with health providers often facing punishment or criminalization for negligence, threats, and violence. Across contexts, successful implementation was driven by integrating MPDSR within quality improvement efforts, improving community involvement, and adapting programming fit-for-context.

**Conclusions:**

The unique contextual considerations of humanitarian settings call for a customized approach to implementing MPDSR that best serves the immediate needs of the crisis, aligns with stakeholder priorities, and supports health workers and humanitarian responders in providing care to the most vulnerable populations.

**Supplementary Information:**

The online version contains supplementary material available at 10.1186/s13031-024-00607-3.

## Introduction

Maternal and perinatal death surveillance and response (MPDSR) and related death review interventions embed quality improvement within mortality surveillance to mount actionable and contextualized solutions to improve care and avert death. Variations of a four-step process or cycle are typically followed using facility- and community-based approaches, including: 1. Identification of notification of deaths; 2. Reporting of deaths; 3. Death reviews, audits, or verbal autopsies with case analysis of the cause of death, social determinants, and contributing health system factors and development of recommendations; and 4. response or implementation of targeted actions to address identified issues.

Implementation of MPDSR and related death review interventions has significantly evolved over the past two decades, catalyzed by World Health Organization’s (WHO) pioneer 2004 guidelines, “Beyond the Numbers,” that recommended all countries implement maternal death reviews to improve the provision of maternal health care [1]. More recently, the Every Newborn Action Plan (2014) and Strategies toward Ending Preventable Maternal Mortality (2015) initiatives have advocated for the institutionalization of more comprehensive mortality review interventions as a strategy to achieve global maternal and neonatal mortality reduction targets by 2030 [2–4]. Guidelines to implement the full cycle and various components of MPDSR, including maternal death surveillance and response (MDSR), and the audit and review of stillbirths and neonatal deaths, have been developed [1, 5–7], and to date, many low- and middle- income countries (LMICs) have adopted such national-level policies and guidelines [8]. Yet, a growing body of evidence suggests few countries are implementing the full cycle at national scale due to a myriad of implementation challenges [9–12].

At the time of writing, more of the world’s population is impacted by armed conflict, extreme weather events, and complex emergencies than ever before. Health systems are strained, and there are United Nations (UN) humanitarian assistance appeals to raise funds for essential services and support to populations in more than 60 LMICs [13]. WHO’s most recent guidelines on MPDSR [7] elevates considerations for implementing MPDSR in humanitarian contexts, including high-level recommendations based on the phase of humanitarian response. In alignment with the Minimal Initial Services Package for Reproductive Health in Crisis Situations (MISP) [14], WHO guidelines emphasize prioritizing immediate health and safety needs during the acute phase (i.e., initial response to an emergency or conflict) to prioritize the delivery of essential services. Once essential maternal and neonatal health services are in place, WHO guidelines suggest that implementation of MPDSR may be considered alongside efforts to strengthen health systems and improve quality of care [7].

Although humanitarian crises are not explicitly documented as a barrier to scale of MPDSR, many of the countries where piecemeal or stagnating implementation has been documented are affected by protracted subnational and/or regional crises [15–21]. Insights from humanitarian practitioners at a global expert consultation in 2019 suggested that many obstacles found within such crisis-affected settings are similar to MPDSR implementation experiences documented in LMICs, albeit exacerbated [16]; however, literature on MPDSR implementation in humanitarian contexts is limited to a few specific programmatic experiences [22]. As global humanitarian needs continue to grow [13], there is a critical need for evidence from these settings on effective methods to collect, analyze, and act on maternal and perinatal mortality data [16, 22, 23].

This study was undertaken on behalf of the global MPDSR Technical Working Group’s sub-group dedicated to MPDSR in Humanitarian Settings (“the SWG”) to understand the complexities of facility-based MPDSR implementation, including how contextual factors influence MPDSR interventions within five humanitarian contexts.

## Methods

### Study design

A descriptive comparative case study was conducted within five varied humanitarian contexts: Cox’s Bazar (CXB) Rohingya refugee camps in Bangladesh, Ugandan refugee settlements, South Sudan, Palestine, and Yemen. Semi-structured individual (*n* = 55) or group key informant interviews (*n* = 11) were conducted from December 2021-July 2022 based on key informant availability and triangulated with case-specific MPDSR documentation obtained through desk review. The study protocol was reviewed by the Johns Hopkins Bloomberg School of Public Health Institutional Review Board and deemed not human subjects research. All study procedures have conformed to the principles embodied in the Declaration of Helsinki.

Cases were defined as specified humanitarian contexts with a 2021 UN humanitarian or refugee response plan with one or more reported MPDSR interventions reported in a recent programmatic landscape analysis conducted by the SWG [24]. We chose to consider the following as ‘MPDSR and related death review interventions’: 1.) maternal, perinatal, or neonatal mortality surveillance systems (e.g., MPDSR, Maternal Death Surveillance and Response (MDSR), Perinatal Death Surveillance and Response (PDSR), Neonatal Death Surveillance and Response (NDSR), and other surveillance systems that document maternal or perinatal mortality) and, 2.) maternal, perinatal, or neonatal death reviews (e.g., death audits, verbal or social autopsies, confidential inquiries, and other related death reviews). Cases were selected to reflect diverse humanitarian settings. Case selection prioritized variation in geographical region, World Bank fragility classification [25], type of humanitarian setting (e.g., refugee, internally displaced persons (IDP), mixed), and reported MPDSR intervention types. Table 1 outlines key characteristics of each selected case.

**Table 1:** Case characteristics

Characteristics	Bangladesh	Uganda	South Sudan	Palestine	Yemen
**Case Context**					
Case focus	Cox’s Bazar Rohingya refugee camps	Uganda refugee settlements	National	West Bank and Gaza	National
WHO Region	Southeast Asia	Africa	Africa	Eastern Mediterranean	Eastern Mediterranean
World Bank Income Classification FY2023 [26]	Lower middle	Low	Low	Lower middle	Low
World Bank Fragility Classification FY2023 [25]	–	–	Conflict	High Institutional and Social Fragility	Conflict
Consecutive years of humanitarian and/or refugee response plans [27]	7	6	13	21	14
People in need of humanitarian assistance 2023 [28]^a^	1.5 M (1%)	1.5 M (3%)	7.8 M (84%)	2.1 M (41%)	21.6 M (73%)
**National Maternal and Perinatal Health Outcomes**					
Maternal mortality ratio [29]^b^	123 (89–174)	284 (191–471)	1223 (746–2009)	20 (15–26)	183 (120–271)
Neonatal mortality rate [30]^c^	16 (14–18)	19 (13–28)	40 (12–105)	9 (7–13)	28 (13–61)
Stillbirth rate [31]^d^	21 (16–26)	15 (14–16)	26 (16–42)	9 (6–16)	23 (16–34)
**National Maternal and Perinatal Health Policy Landscape** [32]					
National law requires every death to be registered	Yes	Yes	Yes	Data not available	Yes
National policy/law on maternal death notification within 24 h	No	Yes	Yes	Data not available	Yes
National policy/law on maternal death review	Yes	No	Yes	Data not available	Yes
National policy/law on neonatal death review	Yes	Yes	No	Data not available	No
Report having national policy / law on stillbirth review	No	Yes	No	Data not available	No

*Abbreviations*: *FY* Fiscal Year, *WHO* World Health Organization

^a^M: million; Reported as n (% of total population)

^b^2020 United Nations Maternal Mortality Estimation Inter-Agency Group Maternal Mortality estimates reported as number of maternal deaths per 100,000 live births; Values in parentheses represent 80% uncertainty intervals

^c^2021 United Nations Inter-Agency Group for Child Mortality Estimation Neonatal Mortality estimates reported as number of neonatal deaths per 1,000 live births; Values in parentheses represent 95% uncertainty intervals

^d^2021 United Nations Inter-Agency Group for Child Mortality Estimation Stillbirth Rate estimates reported as number of stillbirths per 1,000 births; Values in parentheses represent 90% uncertainty intervals

### Study sample

Purposive and snowball sampling were employed to recruit key informants within each case study. Key informants were eligible to participate if they were 18 + years of age, currently engaged in implementing or providing support to MPDSR interventions at national or subnational (e.g., district or regional) levels, and willing to partake in an audio recorded interview. The research team consulted SWG members and representatives from the Interagency Working Group for Reproductive Health in Crises (IAWG), as well as recent MPDSR programmatic landscape analysis findings, to identify agencies implementing or supporting MPDSR interventions to inform recruitment lists.

An estimated 8–20 participants per case study were projected to achieve saturation in key themes. The final sample consisted of 66 interviews with 76 participants across the five settings (Table 2). Participants represented UN agencies, Ministries of Health, international non-governmental organizations (NGOs), local NGOs, health facilities, and other agencies. Organization and agency representation varied by case, reflecting the diversity of crisis-affected contexts selected for the study.

**Table 2:** Interview Participants

	**1. Cox’s Bazar Rohingya refugee camps**	**2. Uganda refugee settlements**	**3. South Sudan**	**4. Palestine**	**5. Yemen**	**Total**
**Interviews**	13	13	17	9	14	66
**Participants**	13	15	22	11	15	76
**Interview hours**	12	12	16	8	11	59
**Participant representation:**						
-UN Agency	10	4	5	8	5	32
-Ministry of Health	0	1	1	1	4	7
-INGO	2	7	9	0	0	18
-Local NGO	0	0	7	0	1	8
-Facility MPDSR focal point	0	2	0	2	4	8
-CDC	1	1	0	0	1	3

*Abbreviations: CDC* Centers for Disease Control and Prevention, *INGO* International Non-Governmental Organization, *MPDSR* Maternal and Perinatal Death Surveillance and Response, *NGO* Non-Governmental Organization, *UN* United Nations

### Data collection

Interested key informants were scheduled to participate in virtual individual or group semi-structured interviews, to respect participants’ time and busy schedules. MM conducted interviews for each case. At the beginning of each individual and group interview, an informed consent script was read to participants and oral consent obtained. Interviews were conducted virtually via Zoom using a semi-structured interview guide to assess key implementation outcomes: adoption, fidelity, penetration, and sustainability (Table 3) [33]. Interviews lasted 45–90 min and were audio-recorded. Interviews were primarily conducted in English; select interviews with key informants in Yemen were supported by live Arabic interpretation to facilitate participants’ ease in communication.

**Table 3:** Key Implementation Outcomes and Corresponding Thematic Constructs

Implementation Outcomes	Definitions	Thematic Constructs	Salience by setting
Adoption	The uptake of MPDSR interventions from the organizational or implementer perspective – how MPDSR interventions are intended to be implemented	• Governance structures • Policy adoption • Implementation processes • Supportingata systems and tools	All contexts
Penetration	The integration of MPDSR interventions within health systems in humanitarian settings	• Scale of implementation (i.e., implementation phase, implementation level, and geographical coverage) • Positionality within health systems • Interoperability with other systems (e.g., surveillance and health information systems)	Contexts in early-mid (1–5 years) or mid-late (5 + years) implementation stages
Sustainability	The extent to which MPDSR interventions are institutionalized within a health system or humanitarian programming	• Local ownership of MPDSR interventions • Sustained funding streams • Institutionalized capacity	Contexts in mid-late (5 + years) implementation stages
Fidelity	The degree to which MPDSR interventions were implemented as intended, according to local, national, or international guidelines or action plans	• Adherence to MPDSR cycle or implementation processes • Quality of reporting and review • Implementing actor responsiveness	Contexts in early-mid (1–5 years) or mid-late (5 + years) implementation stages

In addition, case-specific desk reviews were undertaken; key informants were requested to share MPDSR national policies and guidelines, external reports, and other relevant documents to triangulate findings. Peer-reviewed literature, humanitarian web-portals (e.g., ReliefWeb and Humanitarianresponse.info), and NGO, governmental entity, and UN agency websites were also sourced for a comprehensive review of information related to implementation of MPDSR interventions within each setting.

### Data analysis

Audio recordings of each interview were transcribed and, when necessary, translated into English by an external company and verified by the study team. Interview transcripts and notes, along with supporting documentation obtained from the desk reviews were uploaded into Dedoose qualitative software (version 8.3.45) for analysis. Thematic content analysis was employed to identify themes within and between case studies. While a priori codes were defined based on study outcomes and constructs (Table 3), a serial iterative process was implemented to develop sub-codes and refine the initial codebook with emergent themes [34].

Case descriptions outlining key findings related to the four implementation outcomes (i.e., adoption, fidelity, penetration, and sustainability) and their associated thematic constructs were generated by synthesizing data collected during the key informant interviews. Findings from the complementary data sources were triangulated with key informant interviews to compare and cross-check descriptions of MPDSR adoption in each and to ensure that the case descriptions accurately reflected implementation realities [35]. Key analytical techniques, such as pattern matching and chronological sequencing, were also employed [35]. Preliminary case descriptions were shared with relevant key stakeholders for debriefing and respondent validation.

Cross-case synthesis employed both deductive and inductive analysis. Cross-cutting implementation complexities were identified by examining commonalities and differences in implementation experiences [35]. In addition, within-case patterns or phenomena and convergence or divergence with pre-determined programmatic assumptions (e.g., report and review of deaths will inform response, which in turn, will improve MNH programming) were assessed.

## Results

### Case descriptions

Each case represents a unique implementation landscape for MPDSR interventions in humanitarian settings, with varied implementation phases from small-scale pilots reported in Yemen and South Sudan to mid- to late-phase implementation within Uganda and Palestine (Table 4). Within CXB, a complex multi-partner MDSR system was implemented across the 34 refugee camps. In Uganda refugee settlements, MPDSR serving both refugee and host communities was implemented primarily by United Nations High Commissioner for Refugees- (UNHCR) supported partners and integrated within the national MPDSR system. South Sudan is characterized by fragmented, partner-specific implementation of MPDSR, MDSR, and maternal death review interventions across varying settings, at different implementation phases. In contrast, within Palestine, MPDSR interventions were better defined, albeit also siloed in their approach with partner-specific programming; MDSR and NDSR systems were led and coordinated by the Ministry of Health (MOH) and the United Nations Relief and Works Agency for Palestine Refugees in the Near East (UNRWA) was implementing pregnancy surveillance of refugees seeking care within their primary health centers with subsequent maternal death investigations. Within Yemen, MDSR was implemented within two specified pilot contexts: 1.) MDSR in three districts in Hadhramaut and 2.) MDSR in two tertiary hospitals in Sana’a.

**Table 4:** Case descriptions: implementation landscape of MPDSR interventions

	**1. Cox’s Bazar Rohingya refugee camps**	**2. Uganda refugee settlements**	**3. South Sudan**	**4. Palestine**	**5. Yemen**
Reported intervention types	• MDSR	• MPDSR	• Multiple MPDSR, MDSR, and maternal death review interventions	• MDSR • NDSR • Pregnancy surveillance systems with maternal death investigations	• Two MDSR systems
Implementation phase	• Early-mid phase	• Scaled nationally, including all refugee settlements	• Varies, most in pilot or early implementation phase	• Varies, most mid-large phase	• Pilot phase
Setting	• Refugee Camps	• Refugee settlements	• MPDSR: Refugee camps, urban, and rural • Death reviews: Rural	• MDSR: Urban and rural • NDSR: Urban and rural • Pregnancy surveillance and death investigation: Refugee camps, urban, and rural	• Pilot 1: Urban and rural • Pilot 2: Urban
Population served	• Refugees	• Refugees and host communities	• Refugees, IDPs, and host communities	• Refugees and host communities	• Host communities and IDPs
Health facility level	• All (primary to tertiary)	• All (primary to tertiary)	• Varies by partner	• MDSR/NDSR: Secondary and tertiary hospitals • Pregnancy surveillance and maternal death investigation: Primary health centers and referral secondary and tertiary hospitals	• Pilot 1: Secondary and tertiary hospitals • Pilot 2: Tertiary hospitals

*Abbreviations*: *IDP* Internally Displaced Persons, *MDSR* Maternal Death Surveillance and Review, *MPDSR* Maternal and Perinatal Death Surveillance and Review, *NDSR* Neonatal Death Surveillance and Review

### Implementation outcomes

#### Adoption: how MPDSR was intended to be implemented

The uptake of interventions from the programmatic perspective, otherwise known as adoption, was assessed within each case (Additional File 1) via key constructs including governance structures, policy adoption, implementation processes and supporting data systems and tools.

In 4 of 5 cases, UN agencies had primary or leading roles in initiating, supporting, and coordinating implementation of MPDSR interventions. Implementation was mainly executed by international and local NGOs under UNFPA and UNHCR leadership, with involvement of WHO, United Nations Children’s Fund (UNICEF), UNRWA (Palestine), and International Organization for Migration (IOM; Cox’s Bazar), and support from multi-partner coordination groups in select cases. Recent MPDSR policy and guidelines were only in place in Uganda [36]. MDSR pilots in Yemen were guided by 2013 Yemen Maternal Mortality Audit Guidelines [37], although limited in scope. UNHCR, WHO, and UNFPA technical guidelines were used for partner-specific interventions or to inform development of national procedures.

Implementation processes were largely aligned with the WHO four-step MPDSR cycle [7]. Establishment of review committees occurred in each setting. All cases, with the exception of Yemen, have or were in the process of establishing a national or centralized committee tasked with coordinating implementation of the system, reviewing cases, and/or mounting responses to identified issues. Sub-national committees were also reported in Uganda, South Sudan, and Palestine. All but CXB reported the establishment of review committees or informal teams at the facility level.

Supporting data systems and tools varied widely by case and implementing partner, with parallel reporting systems identified in Uganda, South Sudan, and Palestine (Table 5). In CXB, UNHCR tools were adapted and WHO’s Early Warning Alert and Response System (EWARS) [38] was adopted for maternal death reporting. In Uganda, the MOH integrated an MPDSR event tracker with active death notification and review forms into the District Health Information System 2 (DHIS2), which is further supported by a pilot of UNICEF and Centers for Disease Control and Prevention (CDC) monitoring tools. In addition, maternal and perinatal deaths were reported through the UNHCR system. In South Sudan, tools and reporting systems varied by implementing partner; however, parallel reporting to the MoH and partners often employed DHIS2, the Integrated Disease Surveillance and Response (IDSR) systems [39], UNHCR systems, and other partner-specific systems. In Palestine, each intervention reported through their respective partner-specific system. In particular, UNRWA had implemented a sophisticated electronic health reporting and record system at primary health facilities with a patient smart phone app to support pregnancy surveillance and maternal death investigation and reported through their system and to the MoH. In Hadhramaut, Yemen, a customized electronic system was developed, however its use was discontinued due to funding shortages. In Sana’a, Yemen, UNFPA’s Reproductive Health (RH) logistic management system was linked to maternal death reporting in the absence of a national health information system.

**Table 5:** Key surveillance systems integrated with MPDSR interventions in humanitarian settings

System	Description
Early Warning Alert and Response System (EWARS)	EWARS is “a system that provides an early warning of acute public health events and then connects this function to an immediate public health response. It is one of the most immediate and important functions of a surveillance system. EWARS encompasses the following components and processes: • Early warning – the rapid detection of signals that may indicate potential acute public health events. Sources of early warning data may include notifications from health facilities, community members and other entities, which feed into IBS and EBS systems • Alert management – the systematic process of managing all incoming information, from signal verification to risk assessment and characterization, in order to decide if a response is required to mitigate the public health risk. For efficiency, all signals should preferably be channeled into a common system so that they can be investigated and managed systematically • Response – public health actions triggered by the detection of an alert.” [38]
Integrated Disease Surveillance and Response (IDSR)	IDSR is an “approach for improving public health surveillance and response for priority diseases, conditions and events at community, health facility, district, and national levels. IDSR promotes rational and efficient use of resources by integrating and streamlining common surveillance activities and functions. The IDSR strategy makes surveillance and laboratory data more usable and helps public health managers and decision-makers to improve detection and response to the leading causes of illness, death, and disability in African countries. As part of improvement to the health care system, the IDSR strategy also assisted countries to better monitor and track planned, time-bound targets.” [39]

#### Penetration: scale and integration of MPDSR interventions within health systems

**Fig. 1:**
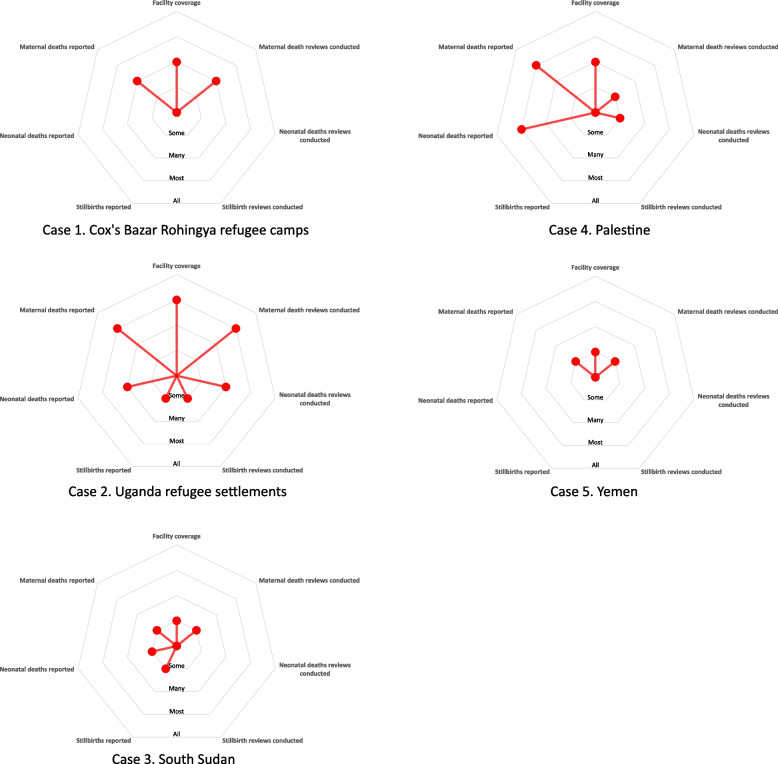
Implementation scale, by case. Figure 1 represents a qualitative appraisal of the scale of maternal death, neonatal death, and stillbirth reporting and review within each case. Each radius represents a component of the MPDSR intervention including health facility coverage, reporting of facility-based deaths, and review of facility-based deaths. The length of each radius is proportional to key informants’ perceptions of the implementation scale of each component using a qualitative ranking of none, some, many, most, and all

The scale of MPDSR interventions within each case differed (Fig. 1) but underreporting of maternal and perinatal mortality occurred across all cases, albeit to varying degrees. Of note, perinatal deaths were more likely to be both underreported and not reviewed compared with maternal deaths. The implementation scale was largest in Uganda, where maternal death reporting and review was well-established, but neonatal death and stillbirth reporting and review only occurred in some facilities. In comparison, the scale of implementation in South Sudan was considerably smaller than in the other four settings: only some facilities were covered by the various reported MPDSR interventions, and limited maternal and no perinatal verbal autopsies were being conducted. In Palestine, where strong national MDSR and NDSR reporting systems were established, most facility-based deaths were reported; however, many Safe Motherhood Emergency Centers located in vulnerable areas in West Bank had not adopted any MPDSR interventions due to limited resources.

While different approaches were undertaken to adopt and scale MPDSR, stakeholders in CXB, Uganda, and some partners in South Sudan aligned or situated implementation of MPDSR interventions within quality improvement (QI) initiatives. Positionality within QI was reported to improve buy-in of MPDSR, increase implementing actor responsiveness, and/or provide inherent accountability mechanisms to mount appropriate responses to identified recommendations to improve care. Alignment with other systems was limited, with the exception of successful integration of humanitarian-led interventions within the national government-led MPDSR system in Uganda. MDSR in CXB had no linkage with Bangladesh’s national MPDSR system, and established national systems did not exist in South Sudan and Yemen. Palestine was also marked by the absence of a harmonized system; attempts to integrate MPDSR and related death review interventions led by the public health system, private sector, NGO sector, and UNRWA under one unified system had yet to be fruitful. Interoperability with other surveillance and health information systems was also rare across the five case studies. Only in CXB and Uganda were MPDSR interventions linked to or integrated with other surveillance systems; WHO’s EWARS and the Integrated Disease Surveillance and Response (IDSR) systems, respectively. In Palestine and Yemen, mortality reporting through the Civil Registration and Vital Statistics systems were noted as opportunities for integration, albeit no established linkages had been reported to date.

#### Sustainability: how MPDSR interventions are institutionalized

The extent to which MPDSR interventions are institutionalized within a health system or humanitarian programming was conceptualized into three primary constructs: sustained funding streams, local ownership of MPDSR interventions, and institutionalized capacity. While assessment of sustainability is most salient for contexts in mid- to large-scale implementation stages, related themes were identified across all cases.

Resources for sustained implementation were not available in any case. While refugee camps and settlements had comparatively more financial and human resources for MPDSR interventions compared to other humanitarian contexts and UNHCR-led programs were able to sustain programming for longer periods of time, all partners reported funding limitations, without dedicated resources for training, capacity building, and response to review findings. MPDSR interventions also did not escape the stark health workforce realities in humanitarian settings; severe shortages and high attrition of health workers, in particular skilled birth attendants, have challenged efforts to achieve institutionalized capacity to implement MPDSR interventions. Across all cases, such implementation (and much of the health sector programming) was dependent upon external humanitarian support and assistance. Given this strong reliance on external support, local ownership of MPDSR interventions was rare, with the exception of MDSR and NDSR systems led by the Palestinian Ministry of Health.

#### Fidelity: how MPDSR interventions were “actually” put into practice

Implementation adherence to applicable guidelines, quality of reporting and review of deaths, and leadership and implementing actor responsiveness were assessed as signs of fidelity to intended MPDSR intervention processes. In CXB, the MDSR system had seen gradual improvement in the reporting of facility-based maternal deaths (i.e., more partners accepting to report deaths in their facilities through the system and increased sensitivity of surveillance at facilities). Nonetheless, notable delays ranging from days to months in conducting death reviews have been reported. Within the Ugandan refugee settlements, implementing partners reported timely notification and review of facility-based maternal deaths, yet perinatal death notification and review was not consistently implemented and strongly implementing partner dependent. In South Sudan and Yemen, MPDSR fidelity was weak. Maternal and perinatal deaths were not reliably reported or reviewed with many established facility-, sub-national-, and national-level review committees having never convened in practice. However, the pilot in Hadhramaut, Yemen had good fidelity with facility-based reporting of maternal deaths. In Palestine, MPDSR interventions had fairly high fidelity marked by a strong maternal and neonatal death notification and reporting system within the Ministry of Health and UNRWA health systems. Nonetheless, extensive delays, often months long, were reported in collection of case information, review, and analysis of maternal and neonatal deaths. National review committees were also not yet functional. Across all case contexts, fidelity to the “response and action” step of the MPDSR cycle was low; only Uganda had ongoing monitoring mechanisms in place at the national level to ensure coordination and accountability of the MPDSR system.

Overall, the quality of reporting and review of deaths was low across all sites. Incomplete data, poor documentation of patient care, and limited access to records at higher-level facilities was pervasive. In 3 of 5 cases (Uganda, South Sudan, and Palestine), misclassification of stillbirths and neonatal deaths was also a recognized challenge due to layered implementation dynamics. Nonetheless, some partner-specific MPDSR interventions demonstrated higher quality implementation, namely UNHCR-led systems with comprehensive reporting and review of maternal and perinatal deaths in refugee camps (Uganda and South Sudan), and UNRWA’s family team approach to primary health care with comprehensive pregnancy surveillance and maternal death investigation of registered patients (Palestine) – both which tended to have high resourced MPDSR programming.

Across all cases, overburdened and under-paid health providers were often reluctant to participate in the notification and/or review of mortality cases, especially when costs were incurred to travel to a health facility. In fact, review committee responsiveness (i.e., high attendance and fidelity to established meeting frequency) typically decreased as they became more decentralized, with the exception of the national MPDSR committee in Uganda. Facility-based review committee members were most active, yet frequency and participation in these committees varied by facility and their implementation partner(s).

#### Cross-case synthesis of implementation complexities

Across the five cases, implementation of MPDSR interventions was often affected by complex implementation climate and system dynamics—characterized by an environment of variable prioritization, buy-in, engagement, and trust from actors at all levels of the system. In most cases, stakeholder prioritization of MPDSR was perceived to be very low given the numerous competing health priorities in these resource-starved contexts.

*“We have a silent killer where the surveillance system has not been customized to document the trends or the cases of deaths as they occur… Prioritization is a problem, and we are all to blame. Everyone needs to do their best to ensure that this comes up on the health agenda.”* – Key informant in South Sudan

Across the cases, the level of leadership engagement in MPDSR also varied. In Palestine and Uganda, strong leadership from the MOH was bolstered by collaboration with UN agencies, INGOs, and local partners to improve implementation. In CXB, implementation of MDSR was first confronted with resistance by humanitarian agencies working within the camps, but engagement and coordination by the various UN-led working groups had strengthened implementation within the complex web of implementing partners. Government support to MPDSR interventions in South Sudan and Yemen has only been reported within the past few years, so leadership has been largely dependent on UNFPA and WHO to sensitize key stakeholders on the value of MPDSR and renew efforts via pilot MPDSR programming.

Regardless of the context, external influence from donors or bi-lateral agencies has been the reported impetus for MPDSR implementation in the study cases, giving legitimacy to the issue and nudging governmental authorities and local stakeholders towards buy-in and support. As a result, the MOHs in many cases were actively developing and/or renewing MPDSR policies, guidance, curricula, and implementation plans. However, partners across the cases expressed disquietude in the conflicting donor priorities and metrics for success – namely, the need to demonstrate active implementation of MPDSR via increased reporting and review of maternal and perinatal deaths to their donors, which simultaneously denoted higher maternal and perinatal mortality, an indicator of poor performance of their health service programming. Reliant upon short-term funding that generally did not allow for long-term sustained systems improvements, many partners, especially local organizations, admittedly did not fully implement MPDSR interventions for this reason. In addition, UN agency influence and mandate often drove MPDSR implementation in often conflicting or disparate approaches.

*“It depends on which agencies are leading, if WHO is leading, they will be asking only ‘What is the maternal mortality ratio?’ because they want to bring the data. But if you ask UNFPA, they will be looking into the actions, ‘What are the actions? What are the recommendations?’ because they want to improve the services there. If you ask UNHCR, they will be only talking about the third delay, quality of care, quality of care, quality of care. So, it’s very contextualized…”  *– Key informant in Cox's Bazar, Bangladesh

Dynamics between implementing actors and perceived implications of the MPDSR intervention also interplayed within the implementation climate. In all contexts, blame culture was pervasive; health providers feared getting blamed, shamed, fined, and/or fired due to their involvement in a maternal or perinatal death case. South Sudan and Uganda, in particular, were reported to have had long histories of politicizing maternal deaths and criminalizing health providers for perceived negligence. In South Sudan, ethnic discord also exacerbated the mistrust in the health system. On numerous occasions, family or community members have been reported to threaten and/or harm providers as a means of retribution for their loss.

*“Sometimes people, if they hear that their relative or a death is because of negligence or delay from the health facility, they will attack and carryout revenge killing in the health facility… A mother … was brought to the hospital due to ruptured uterus…[and] needed a blood transfusion, but there was no blood to transfuse, so the mother passed away. The husband, who was a soldier came with a gun and shot staff. He killed two health workers and injured three. So, in most cases health workers are afraid to give accurate information on what happened to defend themselves from all sides.” –* Key informant in South Sudan

In addition, power dynamics between facility personnel often compelled the omission of facts and secrecy of events, especially when provider errors and mismanagement of a patient occurred. Breached confidentiality during death reviews further fueled mistrust between health providers and death review committee members and bred blame within communities. Buy-in from facility administration was essential to combat blame and mistrust and reorient the implementation climate; they were often gatekeepers for MPDSR with power to set the tone (i.e., supportive learning environment for QI vs punitive process) and ensure consistent participation.

## Discussion

The comparative case study illuminates the complexities of implementing MPDSR interventions in five crisis-affected contexts. By purposively selecting cases that vary in level of insecurity, population served, and programmatic landscape, the study highlights a spectrum of implementation realities and adaptive strategies employed by humanitarian and governmental partners supporting and/or directly performing MPDSR interventions. Adoption, penetration, sustainability, and fidelity to established MPDSR interventions varied widely. Nonetheless, our study offers the first comparative analysis of key obstacles, drivers of implementation success, and best practices across humanitarian contexts. Table 6 summarizes key lessons learned.

**Table 6:** Key takeaways: Complexities of implementing Maternal and Perinatal Death Surveillance and Response in crisis-affected contexts

1. Scaling MPDSR interventions in low- and middle-income countries requires consideration of additional complexities and actors in humanitarian contexts.
2. Limitations in financial and human resources underscore all MPDSR implementation challenges in crisis-affected contexts.
3. UN agencies and implementing partners influence MPDSR implementation with often conflicting or disparate priorities and approaches within the same context.
4. Variable prioritization, buy-in, and engagement by actors at all levels impedes successful implementation of MPDSR interventions.
5. Reporting and reviewing maternal and perinatal deaths often create an environment susceptible to blame, which may be further fueled by community tensions and ethnic discord in humanitarian settings.
6. Customization of each step of the MPDR cycle is essential for optimal functionality in humanitarian settings along with health systems approaches that account for complex implementation climates.

Similar to other published implementation experiences in humanitarian settings [22], our findings underscore the significant challenge of implementing MPDSR interventions with limited funding and personnel. While some partners have mitigated issues using stop-gap measures, sustained implementation will be dependent upon dedicated funding to support activities such as review meetings, refresher training, and response plans. Furthermore, implementation plans should take into account and adjust expectations vis-à-vis health worker realities in fragile and humanitarian contexts – undervalued health workers with little resources within insecure settings may not be motivated by MPDSR’s inherent advantage of preventing death, nor committed or willing to ‘self-correct’ for the sake of learning [10, 40, 41] until adequately compensated for their already burdensome workload.

Thoroughly documented in LMICs [9], MPDSR implementation in humanitarian settings is also fraught with a precarious implementation climate susceptible to blame, which may be further fueled by community tensions and ethnic discord. Efforts to establish MPDSR interventions within a framework of learning and QI have been reported, with many of Kinney et al.’s (2022) micro- and meso-level strategies undertaken by partners to overcome blame culture across cases [42]. Nonetheless, given reported societal politicization and criminalization of deaths, realizing a ‘no blame, no shame, no name’ culture for MPDSR will not be feasible in many humanitarian contexts until supportive MPDSR legislation and national policies are enacted and promulgated to safeguard all actors. Many identified implementation complexities and system dynamics were influenced by the blame culture, calling for nuance, prescience, and attention to the implementation climate when developing and/or supporting MPDSR interventions within these contexts.

Given the challenges of implementing programs in resource-poor settings, some colleagues have debated the value-added of MPDSR and advocate instead for dedicating already-limited resources to programming focusing on known drivers of high maternal and neonatal mortality [10]. Current guidelines support the prioritization of establishing essential maternal and neonatal health services in acute humanitarian settings before considering the implementation of MPDSR [7].Nonetheless, taking into account implementation dynamics in early evidence-based and context-specific adaptations of MPDSR guidelines will maximize successful implementation in humanitarian contexts fraught with severe resource shortages and the highest maternal and neonatal mortality burden. Our findings suggest customization of each step of the cycle is essential for optimal functionality in humanitarian settings – along with health systems approaches that account for complex implementation climates that will influence implementation fidelity. Additional implementation research is needed to better understand how to adapt and sustain MPDSR implementation, especially within contexts of fluctuating insecurity.

The complexity of the stakeholder landscape within humanitarian contexts must be accounted for. Each implementing agency and partner’s approach and mandate for MPDSR interventions, albeit mortality estimation, quality improvement, both, or somewhere in-between, significantly influences implementation on the ground, creating islands of programming varying in scope, quality, and reach. Coordination and alignment of MPDSR programming across partners can positively impact adoption and fidelity within humanitarian settings. Similarly, integration with existing national systems, strategies, and policies serves to improve care across all populations in the affected country.

In addition, the global recommendation to initiate MPDSR programming at tertiary health facilities [6] may need to be reconsidered in some crisis-affected contexts, where a vast majority of deliveries occur at home and community-based health delivery strategies are in place to compensate for weakened health systems; in these settings, starting with community-based MPDSR approaches may provide more insights on how actors can avert deaths and establish programming where formal health systems are fractured or not functioning [15, 17–19, 21, 43–47]. Some research posits that MPDSR may be underutilized in crisis-affected contexts due to challenges in identifying maternal and perinatal deaths, especially when most occur outside of the formal health system [48–50]. Future research investigating the effectiveness of community-based MPDSR approaches in humanitarian settings could greatly contribute to the evidence base.

The comparative case study had several limitations. Due to the specificity of each case context, findings may lack external validity to populations outside the five selected humanitarian settings. Nonetheless, identified cross-cutting programmatic dynamics are informative for humanitarian settings globally. In addition, due to travel restrictions compelled by the COVID-19 pandemic, all interviews were conducted via Zoom in English, with the support of live Arabic interpretation when needed. Online interviews may have limited the sample of key informants to those with access to the internet and impacted the dynamics of the discussions (e.g., the interviewer was unable to document/acknowledge nonverbal cues during the interviews). To adapt to key informants’ busy field schedules, group interviews were also offered as an alternative solution. Depending on who participated, power dynamics may have biased participants’ responses in group interviews. Similarly, given MM’s global north background, unequal power dynamics between the interviewer and participant could have been possible leading to social desirability bias. To minimize these effects, MM encouraged balanced participation and contribution from participants and practiced ongoing reflexivity. Lastly, since transcription and analysis were conducted by a non-local researcher (MM), socio-cultural nuances may have been missed; to minimize any misinterpretation, study result validation was undertaken with key informants from each case.

## Conclusions

The unique contextual considerations of humanitarian settings call for a customized approach to implementing MPDSR interventions that best serves the immediate needs of the crisis, aligns with stakeholder priorities, and supports health workers in providing care to the most vulnerable populations. Improved coordination and alignment of MPDSR programming across humanitarian partners and government actors is crucial for scale-up within LMICs. Further development of global guidance should consider the implementation complexities of humanitarian settings and address how to introduce, scale-up, and sustain MPDSR implementation within fluctuating crises to effectively contribute to maternal and perinatal mortality reduction globally.

### Supplementary Information

Additional file 1. Implementation outcomes summarized by case. Key study implementation outcomes are summarized by case.

## Data Availability

No datasets were generated or analysed during the current study.

## Abbreviations

CDC: Centers for Disease Control and Prevention
CHW: Community health worker
CRADA: Christian recovery and development agency
CRVS: Civil Registration and Vital Statistics
CXB: Cox’s Bazar
DHIS2: District Health Information System 2
EPI-WG: Epidemiology working group
EWARS: Early Warning Alert and Response System
FH/MTI: Food for the Hungry/Medical Teams International
GNN: Gaza neonatal network
IAWG: Interagency Working Group for Reproductive Health in Crises
IDP: Internally displaced persons
IDSR: Integrated Disease Surveillance and Response
IMC: International medical corps
IOM: International Organization for Migration
IP: Implementing partner
IRC: International rescue committee
LMIC: Low- and middle- income country
MDSR: Maternal Death Surveillance and Response
MIHR: Momentum Integrated Health Resilience
MNH: Maternal and Neonatal Health
MOH: Ministry of Health
MoPHP: Ministry of Public Health and Population
MPDSR: Maternal and Perinatal Death Surveillance and Response
NDSR: Neonatal Death Surveillance and Response
NGO: Non-Governmental Organization
PDSR: Perinatal Death Surveillance and Response
PHD: Partners in health and development
QI: Quality improvement
RH: Reproductive health
RMNCH: Reproductive maternal neonatal and child health
RTMI: Research, training and management international
SAVE: Save the children
SRH: Sexual and reproductive health
SWG: Sub-working group
TADO: Touch Africa development organization
ToT: Training of trainers
UN: United Nations
UNFPA: United nations population fund
UNHCR: United Nations high commissioner for refugees
UNICEF: United Nations children’s fund
UNRWA: United Nations Relief and Works Agency
WHO: World Health Organization
WRA: Women of reproductive age
