# Supplementary material for: Complexities of implementing Maternal and Perinatal Death Surveillance and Response in crisis-affected contexts: a comparative case study
Source: Confl Health. 2024 Jul 16;18:45. doi: 10.1186/s13031-024-00607-3 (PMC11251288; doi:10.1186/s13031-024-00607-3)
Supplement: Supplementary file 1 — Additional file 1. Implementation outcomes summarized by case. Key study implementation outcomes are summarized by case. [file 13031_2024_607_MOESM1_ESM.docx]

**Additional File 1: Implementation outcomes summarized by case**

|  | **1. Cox’s Bazar Rohingya refugee camps** | **2. Uganda refugee settlements** | **3. South Sudan** | **4. Palestine** | **5. Yemen** |
| --- | --- | --- | --- | --- | --- |
| **Adoption:** The uptake of MPDSR interventions from the organizational or implementer perspective- how MPDSR interventions were intended to be implemented | | | | | |
| **Governance structures** | - UN agencies: UNFPA, UNHCR, WHO, IOM - IPs: PHD, RTMI, FH/MTI, Hope Foundation - SRH and EPI Working groups | - MoH - UN agencies: UNHCR, UNFPA, UNICEF - IPs: IRC, CARE, AVSI, Medical Teams International, African Humanitarian Action | - MoH - UN agencies: UNFPA, WHO, UNHCR, UNICEF - INGOs and IPs: CARE, SAVE, IMA, IMC, MIHR, GOAL, AMREF, CORDAID, TADO, CRADA, etc. | - MDSR and NDSR led by MoH and supported by UNFPA, WHO, UNRWA, Gaza Neonatal Network (GNN) - Pregnancy surveillance system with maternal death investigations led by UNRWA | - MoPHP in North and South - UN agencies: WHO, UNFPA, UNHCR, UNICEF |
| **Policy adoption** | - No policies or guidelines established - Standard operating procedure developed | - 2008 Presidential decree declared maternal death a notifiable condition - 2017 MOH adopted WHO MPDSR guidelines - UNHCR guidelines used in many settlements | - No current MOH MPDSR guidelines - UNFPA and UNHCR guidelines and protocols used by many partners | - No policies or guidelines established at national level - MoH developed a Terms of Reference for maternal death review committees - UNRWA technical guidelines and instructions | - 2013 Maternal Mortality Audit National Guidelines |
| ***Implementation processes*** | | | | | |
| **Facility-based processes** | - Follows 4-step cycle | - Follows 4-step cycle | - Varies, most partners follow 4-step cycle | - MDSR/NDSR follow 4-step cycle - Pregnancy surveillance: follow up status of pregnant patient via family or health facility and conduct maternal death investigation, if case identified | - Both pilots follow 4-step cycle |
| **Review committees** | - MPDSR committee and sub-committee | - National, district, and facility-based committees | - Varies by partner and location: National, state, county health department, and facility committees | - National committee: newly conceived, not active - Subcommittees for Gaza and West Bank - National neonatal mortality committee & GNN committee - Informal facility-based teams | - Pilot 1: Governorate and district review committees - Pilot 2: Governorate investigation teams |
|  |  |  |  |  |  |
| ***Implementation readiness*** | | | | | |
| **Data systems & Tools** | - WHO’s Early Warning Alert and Response System (EWARS) - Adapted UNHCR tools | - Dual reporting systems: UNHCR and MoH DHIS2 - MoH DHIS2 with event tracker and live MPDSR death notification and death review form - Monitoring tools being piloted by UNICEF and CDC | - Dual reporting systems: DHIS2, IDSR, partner-specific systems - Tools vary by partner | - Parallel partner-specific systems - MoH questionnaire (recently updated) - UNRWA tools and systems: e-health reporting and record system, smart phone apps, investigation report, and full report | - Pilot 1: Customized e-system used for 6 months – discontinued due to funding shortages - Pilot 2: UNFPA RH Logistic management information system linked to maternal death report - Adapted WHO forms |
|  |  |  |  |  |  |
|  | | | | | |
|  |  |  |  |  |  |
|  |  |  |  |  |  |
|  |  |  |  |  |  |
| **Penetration:** The integration of MPDSR interventions within health systems in humanitarian settings | | | | | |
| **Positionality within health system** | - No alignment with national system - Gradual shift from counting deaths to leveraging MDSR as QI tool | - Strong integration within national MPDSR system - MPDSR situated within broader QI framework | - No national system - Many partners integrate MPDSR within RMNCH services and nutrition programming to improve sustainability - Some partners align MPDSR with QI processes | - Established national systems but Safe Motherhood Emergency Centers (10) located in most vulnerable areas in West Bank not yet reporting cases - Absence of a harmonized system: multiple attempts to integrate MoH, private sector, NGO sector, and UNRWA under one common system | - Siloed systems with little integration with other programs |
| **Interoperability with other surveillance systems** | - Integrated within WHO EWARS | - Maternal death integrated into IDSR as notifiable condition | - None reported | - Mortality reporting through CRVS not linked to MDSR/NDSR | - Newly adopted legislation on reporting of maternal and neonatal mortality through CRVS – yet no established linkages |
| **Sustainability:** The extent to which MPDSR interventions are institutionalized within a health system or humanitarian programming | | | | | |
| **Sustained funding streams** | - None | - Sustained funding from UNHCR | - None | - Limited funding for implementation | - None |
| **Local ownership of MPDSR interventions** | - None: coordinated by UNFPA, UNHCR, WHO and working groups | - Limited: alignment with national system but managed by UNHCR | - Limited: MoH pilot facilities are supported fully/mostly by external partners | - Strong MoH leadership and ownership of MDSR/NDSR | - Limited: recent buy-in from MoPHP in North and South |
| **Institutionalized capacity** | - None | - Institutionalized mentorship and training | - None | - Key MoH leadership with technical skills and capacity to implement | - None |
| **Fidelity:** The degree to which MPDSR interventions were implemented as intended, according to local, national, or international guidelines or action plans. | | | | | |
| **Adherence to MPDSR intervention cycle or processes** | - Gradual improvement in reporting of facility-based maternal deaths - Variable implementation of response | - Timely notification and review of facility-based maternal deaths - Ad hoc implementation of perinatal component of MPDSR – partner dependent - Variable implementation of response | - Maternal and perinatal deaths not consistently reported through MoH channels - Many review committees established but have never convened - Response needs strengthening | - Strong maternal and neonatal death notification and reporting system - Extensive delays between collection of case information, review, and analysis of maternal and neonatal deaths at national level - National review committees are not active - Facility-based committees lack accountability mechanisms - Response needs strengthening | Pilot 1:   - Good reporting at health facilities - Delays in review of facility-based maternal deaths - No formal response component due to financial constraints   Pilot 2:   - Low adherence to implementation cycle - Some reporting occurs - Reviews and response components are not implemented |
| **Quality of reporting and review** | - Triangulation of community and facility-based reporting identifies unreported facility deaths - Incomplete data and poor documentation of patient care - Documentation of referred patient care is very limited | - Incomplete data and poor documentation of patient care - Maternal death reports lack cause of death classification - Misclassification of perinatal deaths - UNHCR reporting tool more comprehensive than national system | - Incomplete data and poor documentation of patient care - Health provider literacy impacts data quality at primary health facilities - Misclassification of perinatal deaths | - Incomplete data and poor documentation of patient care - Unavailability or delays in receiving patient records, especially from private sector - Misclassification of perinatal deaths - UNRWA electronic system provides comprehensive data during antenatal period among registered patients, but gaps when relying on data from other systems | - No universal or national health information system - Incomplete data and poor documentation of patient care - Reported cause of death often incorrect and/or case details vague or misleading - Documentation of patient care during referrals is very limited |
| **Implementing actor responsiveness** | - Few subcommittee members actively participate in reviews - Health providers reluctant to participate | - Very active national MPDSR committee - District and facility committees are more active and functional within settlement districts - Health providers reluctant to participate | - Active national MPDSR committee tasked with developing national system - State committees are not functional - Facility committees more active and functional within refugee camps - Health providers reluctant to participate | - National committees not yet active - Frequency and participation in facility-based committee reviews varies by facility - Health providers demotivated to participate due to high workloads, limited salaries, and health system constraints | Pilot 1:   - Few members actively volunteer on review committees - Health providers actively participate and report deaths   Pilot 2:   - Investigation team not active/functional - Health providers demotivated and not participating due to high workloads and limited/no salaries |
| *Notes:*  *Abbreviations: CDC: Centers for Disease Control and Prevention; CHW: Community Health Worker; CRADA: Christian Recovery and Development Agency; CRVS: Civil Registration and Vital Statistics; DHIS2: District Health Information System2; EPI: Epidemiology; EWARS: Early Warning Alert and Response System; FH/MTI: Food for the Hungry/Medical Teams International; GNN: Gaza Neonatal Network; IDSR: Integrated Disease Surveillance and Response; IMC: International Medical Corps; IOM: International Organization for Migration; IPs: Implementing Partners; IRC: International Rescue Committee; MDSR: Maternal Death Surveillance and Response; MIHR: Momentum Integrated Health Resilience; MOH: Ministry of Health; MoPHP: Ministry of Public Health and Population; MPDSR: Maternal and Perinatal Death Surveillance and Response; NDSR: Neonatal Death Surveillance and Response; NGO: Non-governmental Organization; PHD: Partners in Health and Development; QI: Quality Improvement; RMNCH: Reproductive Maternal Neonatal and Child Health; RTMI: Research, Training and Management International; SAVE: Save the Children; SRH: Sexual and Reproductive Health; TADO: Touch Africa Development Organization; UN: United Nations; UNFPA: United Nations Population Fund, UNHCR: United Nations High Commissioner for Refugees; UNICEF: United Nations Children’s Fund; UNRWA: The United Nations Relief and Works Agency; WHO: World Health Organization; WRA: Women of Reproductive Age* | | | | | |
